# Supplementary material for: Evaluation of the effectiveness of macaíba palm seed kernel (Acrocomia intumescens drude) on anxiolytic activity, memory preservation and oxidative stress in the brain of dyslipidemic rats
Source: PLoS One. 2021 Mar 17;16(3):e0246184. doi: 10.1371/journal.pone.0246184 (PMC7968719; doi:10.1371/journal.pone.0246184)
Supplement: Attachment — Submitted filename: Silva et al R1 Response to Reviewers.docx [file pone.0246184.s002.docx]

PONE-D-20-18348

MACAÍBA ALMOND (Acrocomia intumescens Drude) CONSUMPTION REDUCES ANXIETY AND COMPROMISE MEMORY IN RAT

Reviewers' comments:

Reviewer #1:

1) Title

The title did not reflect the nature of the study and as seen from the manuscript the effectiveness of macaíba almonds was evaluated on anxiolytic activity, memory preservation and oxidative stress in the brain of dyslipidemic rats.

Hence, the title should read:

- Evaluation of the effectiveness of macaíba almonds on anxiolytic activity, memory preservation and oxidative stress in the brain of dyslipidemic rats.

Reply: Thank you, it was modified.

2) Abstract

The Abstract did not provide an informative and quantitative summary of the research undertaken while the Conclusion was not stated in the Abstract.

Reply: It was inserted: "...Results demonstrated that the animals treated with almonds realized more rearing. DG and AG groomed less compared with CONT and DAG compared with all groups in OFT. AG spent more time in aversive open arms compared with CONT and DAG compared with all groups in EPM. Only DAG spent more time in the central area in EMP. AG and GAD showed a reduction in the exploration rate and MDA values (p <0.05). Data showed that macaíba almond consumption induced anxiolytic-like behaviour and decreased lipids peroxidation in rats' brains. On the other hand, this consumption by healthy and dyslipidemic animals compromises memory.

3) Introduction

The problem statement was not clearly spelt out. A clear justification on the importance of this study should be deliberated.

Reply: It was inserted: "Yet when consumed in excess, lipids can lead to metabolic disorders such as obesity which is associated with brain disorders, and cognitive decline, a manifestation of neurodegenerative disease [8-10]. An experimental study involving administration of palmitic acid, a saturated fatty acid, augmented anxiety-like behavior in animals [11]. Animals fed high-fat diets (HFD) reveal reduced levels of brain-derived neurotrophic factor in the hippocampus, reduced neurogenesis, and impaired learning as compared to animals fed standard diets [12-14]. Changes in hypothalamic-pituitary-adrenal axis functions that comprise cognitive and behavioral functions are often due to excessive consumption of lipids, causing deficiencies in emotional processes and in stress response [15]. However, we do not know if dyslipidemia can induce brain changes and how the consumption of foods source of lipids and antioxidants compounds can reverse possible damage."

4) Materials and Methods

➢ All the chemicals used in the study should be named in this section.

Reply: Thank you.. It was inserted.

5) Results

Results are not well deliberated.

➢ Where is the description for below section in the manuscript:

3.4 Determination of phenolic compounds and total antioxidant activity

Reply: We apologize. It was inserted (Table 1).

6) Discussion

Author need to discuss about the Results obtained rather that used the general sentences. There is no possible explanation for findings and no conclusion with implication for each section of the work. Please revise the relevance of the findings in the light of other comparable studies.

Reply: We improved the writing of the discussion, but we had difficulty finding works that investigated the effects of almond consumption on the nervous system.

We hope it was enough.

7) Conclusion

The Conclusion drawn in this study is not clear nor convincing. Authors should also include in this section: constrains/limitations of the present study and future studies to be undertaken. The conclusion should provide a place to persuasively and succinctly restate the research problem.

Reply: It was modified: " Data showed that the consumption of macaiba almonds reduced lipid peroxidation in brains and induced anxiolytic-like behaviour in health and dyslipidemic rats.

On the other hand, this almond consumption compromised the rats’ learning and memory performance.  To better elucidate this mechanism of memory impairment, future analyzes measuring brain-derived neurotrophic factor and neurotransmitters can be performed."

8) Figures

➢ Figures What is X and Y axis? Each axis should be labeled with name and unit.

Reply: It was modified.

9) English language usage

The poorly worded/construction of the sentences should be improved through the text by a native English speaker to ensure the use of standard and correct English.

10) Please rectify the below throughout the manuscript:

- (e.g. Author stated: .....reflux for 4 min --------a further 3 minutes...Which one is correct?? min or minutes)

Reply: Thank you. It was modified for minutes.

➢ Authors did not adhere to the format of the Journal

Reply: Thank you. It was modified.

➢ Please check for:

[41] (BUDZYNSKA et al., 2015).

[45] (BASIRICO et al, 2017).

Reply: Thank you. It was removed.

Reviewer #2:

1. The article is not written in a proper English language The article should be re-written with the help of English language expert.

2. How did the rat administer with almond. what is almond flour solution. How it was prepared. Add the details.

Reply: Thank you. It was inserted: "To obtain macaiba almond flour, the peel and pulp was removed from the macaíba fruit, chestnut was broken and almonds were removed. After obtaining the almond, the films surrounding the almond were removed and taken to grind to obtain the flour. The flour was taken to a drying oven with air circulation at 55ºC (± 1 ° C) during 24 hour. The flour was sieved to eliminate larger grains; then stored in a vacuum and kept on refrigeration until the moment of use. To prepare the solution, distilled water was used as a vehicle. The solution was made with 0.1% of the macaíba almond flour."

3. Describe the composition of HFE and its method of preparation.

# Reply: Thank you. It was inserted: "Before starting treatment, dyslipidemia according to the methodology adapted of Xu et al. [24] was induced in the dyslipidemic groups, by means of an HFE. To prepare a 420 ml solution (HFE), we used: 168 g of lard, 4.70 g of cholesterol, 32.5 g of pasteurized powdered egg yolk (Salto's ®), 8.4 g of bile acid, 42 ml of glycerol, 4.2 g of propiltiouracil and warm distilled water to complete the volume of 420 ml. The solution was stored in the refrigerator and before use heated in a water bath at 42 ° C daily."

4. It is not clear whether the administration of HFE was continued through out the experimental duration or it was only given before the supplementation of almond for 14 days.

# Reply: Thank you. It was inserted. "The HFE was administered through gavage (at 10 ml/kg of rat weight, once a day, for 14 days prior to the start of treatment. After this period, the animals no longer received HFE."

5. Mention the recorded parameter of test at the end of each test.

# Reply: Thank you. It was inserted.

6. In Object recognition test, the nature of object A1, A2, and A3 is not clear, whether all three were different ot there was any similarity existed between the objects.

Reply: Thank you. To make objects heavy and to prevent locomotion, we fill their interior with plaster.

It was inserted:"Twenty-four hours after the OFT, the object recognition test was performed (using the open field apparatus), to assess the animals' short-term memory. The test consists of first getting the animals used to the open field in the absence of any object, where the can animals freely explore the arena for 10 minutes. Afterwards, in the training session, the animal is placed in the open field with object A1 (familiar object) and object A2 (unfamiliar) where exploration is allowed for 10 minutes. After this training session, at 180 minutes the short-term memory test is performed, where object A1 continues, and object A2 is replaced by object A3 (a new object), and an exploration time of 3 minutes. The animal's exploration time involves sniffing and touching the object with its front legs and/or snout [25]. We used heavy plastic objects with different colors and shapes."

7. The experimental protocol involve two factors; therefore the data should re-analyzed by two-way ANOVA.

Reply: Thank you. It was done.

8. Since authors are explaining compromised memory in term of lipid content of almond, so the lipid content of almond should be analyzed.

Reply: Thank you. It was inserted in Table 2.

9. Merge the small paragraphs to make comprehensive para.

Reply: Thank you. It was done.
