# Supplementary material for: Evaluation of the effectiveness of macaíba palm seed kernel (Acrocomia intumescens drude) on anxiolytic activity, memory preservation and oxidative stress in the brain of dyslipidemic rats
Source: PLoS One. 2021 Mar 17;16(3):e0246184. doi: 10.1371/journal.pone.0246184 (PMC7968719; doi:10.1371/journal.pone.0246184)
Supplement: Attachment — Submitted filename: Silva et al R2 Response to Reviewers.docx [file pone.0246184.s003.docx]

PONE-D-20-18348

MACAÍBA ALMOND (Acrocomia intumescens Drude) CONSUMPTION REDUCES ANXIETY AND COMPROMISE MEMORY IN RAT

Reviewers' comments:

Reviewer #2:

Reviewer #2: Comments
1. In introduction authors are stating “There are few studies assessing the potential and quality of macaíba almonds, and include possible effects of their ingestion” it is better to mention those studies and then mention the purpose of the current study. This would clearly highlight the novelty of the study.

Reply: Thank you. It was removed.

“There are few studies assessing the potential and quality of macaíba pulp and oil [22, 24]. A research confirmed the anti-inflammatory and diuretic activities of the pulp’s oil in rats [25]. However, there is no scientific evidence of the effect in human or animal health induced by the consumption of macaiba almonds.

Based on the above, it is expected that the macaíba almond will present protective and antioxidant effects, with anxiolytic activity and memory preservation in health rats or with dyslipidemia. The aim of this study was to evaluate the effects of macaíba almonds on anxiety behavior and short-term memory in adult health and dyslipidemic Wistar rats.”

2. Mention the source of chemicals

Reply: Thank you. It was inserted.

**Chemicals**

2,2′-Azino-bis(3-ethylbenzothiazoline-6-sulfonic acid) diammonium salt - ABTS (Chemical formula C_18_H_24_N_6_O_6_S_4;_ Sigma); 2,4,6-Tris(2-pyridyl)-s-triazine - TPTZ (Chemical formula C_18_H_12_N_6;_ Sigma); (±)-6-Hydroxy-2,5,7,8-tetramethylchromane-2-carboxylic acid – Trolox (Chemical formula C_14_H_18_O_4;_ Sigma); β-Nicotinamide adenine dinucleotide 2′-phosphate reduced tetrasodium salt hydrate - NADPH (Chemical formula C_21_H_26_Na_4_O_14_P_3_H_2_O; Sigma); Acetone (Chemical formula CH_3_COCH_3_; VETEC ); Aluminum Chloride (Chemical formula AlCl_3_; NEON); Calcium carbonate (Chemical formula  CaCO_3_; NEON); Catechin Hydrate (Chemical formula C_15_H_14_^.^ 6H_2_O; Sigma); Chloroform (Chemical formula CHCl₃; VETEC); DTNB 5,5′-Dithiobis (2-nitrobenzoic acid) (Chemical formula C_14_H_8_N_2_O_8_S_2_; Sigma); Ethanol (Chemical formula C_2_H_5_OH; VETEC); Ether (Chemical formula (C2H5)2O; CLAE J.T. Baker - Plillipsburg, USA); Ferric chloride hexahydrate (Chemical formula  FeCl_3_·6H_2_O; NEON); Folin–Ciocalteau’s phenol reagente (Sigma); Gallic Acid (Chemical formula (HO)3C6H2CO2H; Sigma); Glacial Acetic acid (Chemical formula CH_3_CO_2_H; VETEC); Glutathione reductase (Chemical formula C_10_H_17_N_3_O_6_S ; Sigma); Hexane (Chemical formula C6H14; CLAE J.T. Baker - Plillipsburg, USA); Hydrochloric Acid (Chemical formula HCL; NEON); Methanol (Chemical formula CH4O; CLAE J.T. Baker - Plillipsburg, USA); Potassium hidroxide (Chemical formula KOH; VETEC); Potassium Persulfate (Chemical formula K_2_S_2_0_8;_ NEON); Sodium acetate (Chemical formula CH_3_COONa; NEON); Sodium carbonate (Chemical formula Na2CO_3;_ NEON); Sodium hydroxide (Chemical formula NaOH; NEON); Sodium Nitrite (Chemical formula NaNO_2;_ NEON); Sodium sulfate (Chemical formula Na2SO4; F. MAIA); Trichloroacetic acid –TCA (Chemical formula C_2_HCl_3_O_2;_ Sigma).

3. What are ABTS and TPC? Abbreviations are still not properly defined. Define the abbreviations at the first place and use them consistently.

Reply: Thank you. It was inserted.

“The ABTS (2,2'-Azino-Bis (3-Ethylbenzothiazoline-6-Sulfonic Acid) Diammonium Sal) method was performed according to the methodology described by Surveswaran et al. [30], with modifications.

The absorbance of the extract was compared with a standard curve of gallic acid to estimate the total phenolic compound (TPC)…”

4. The universal unit for the speed of centrifugation is g (gravitational force). Mention all the “rpm” value in “g” values.

Reply: Thank you. It was modified.

MDA: “Then, placed in a water bath, with agitation, at 45 ° C, for 40 minutes and, subsequently, taken to centrifugation at 2500 xg, for 5 minutes, at 4°C.”

Total Carotenoids: “Subsequently, centrifugation was performed at 5724 xg for 10 minutes for later reading using a spectrophotometer (BEL Photonics, Piracicaba, São Paulo, Brazil) at 450 nm.”

Total yellow flavonoids: **“**Afterwards, centrifugation was performed at 7244 xg for 15 minutes, for later reading with a spectrophotometer (BEL Photonics, Piracicaba, São Paulo, Brazil) at 374 nm.”

5. Authors have analyzed the results by two-way ANOVA but they are not mentioning the statistical effect of two factors (dyslipidemia and almond treatment). I would suggest to follow the pattern of writing all results as follow:
Elevated plus maze test (EPM)
The statistical analysis of EPM data by two-way ANOVA showed significant/non-significant effects of dyslipidemia [F(df) = ….., p = …], macaíba consumption [F(df) = ….., p = …], and interaction between dyslipidemia and macaíba consumption [F(df) = ….., p = …]. AG and DAG groups presented decreased entries in the open arms in as compared to the CONT and DG groups (Figure 2A) (p <0.05). DG group also showed reduced number of entries than CONT (p <0.05). Both groups treated with macaiba almond (AG and DAG) spent more time in open arms (p <0.05) as compared to ……???? group (Figure 2B). Consistent with these results, DAG group stayed longer in the central area when compared to all groups (p <0.05) (Figure 2C).

Thank You. It was inserted:

“**Open field test**

   According to the results, DG, AG and DAG showed less ambulation than CONT (Figure 1A). The statistical analysis by two-way ANOVA showed significant effects of dyslipidemia [F(1.24) = 19.94, p =0.0002], no significant effects of macaíba consumption [F(1.24) = 3.372, p = 0.0787], and interaction between dyslipidemia and macaíba consumption [F(1.24) = 10.67, p =0.0033].

Both groups treated with macaiba realized more rearing than CONT and DG (Figure 1B). Two-way ANOVA statistical analysis showed non-significant effects of dyslipidemia [F(1.32) = 1.716, p =0.1995], significant result by macaíba consumption [F(1.32) = 55.38, p < 0.0001], and no interaction between dyslipidemia and macaíba consumption [F(1.32) = 1.582 p = 0.2176].

The grooming was decreased in DG and AG compared to CONT and DAG compared to all groups (Figure 1C). Two-way ANOVA statistical analysis showed significant difference of dyslipidemia [F(1.52) = 29.85, p <0.0001], macaíba consumption [F(1.52) = 38.34, p < 0.0001], and no interaction between dyslipidemia and macaíba consumption [F(1.52) = 1.194 p = 0.2796].

**Elevated plus maze test (EPM)**

AG and DAG groups presented decreased entries in the open arms in as compared to the CONT and DG groups (Figure 2A) (p <0.05). DG group also showed reduced number of entries than CONT (p <0.05). The statistical analysis by two-way ANOVA showed significant effects of dyslipidemia [F(1.44) = 3.953, p =0.05], macaíba consumption [F(1.44) = 92.62, p < 0.0001], and interaction between dyslipidemia and macaíba consumption [F(1.44) = 327.6, p =0.008].

Both groups treated with macaiba almond (AG and DAG) spent more time in open arms (p <0.05) as compared to CONT and DG groups (Figure 2B). The statistical analysis of EPM data by two-way ANOVA showed significant effects of dyslipidemia [F(1.52) = 210.5, p < 0.0001], macaíba consumption [F(1.52) = 1257, p < 0.0001], and interaction between dyslipidemia and macaíba consumption [F(1.52) = 327.6, p < 0.0001].

Consistent with these results, DAG group stayed longer in the central area when compared to all groups (p <0.05) (Figure 2C). Two-way ANOVA statistical analysis showed significant effects of dyslipidemia [F(1.44) = 27.92, p < 0.0001], macaíba consumption [F(1.44) = 14.5, p = 0.0004], and no interaction between dyslipidemia and macaíba consumption [F(1.44) = 0.8694 p =0.3562].

**Object recognition test**

As for the object recognition test, a reduction in the rate of exploration of the new object was observed in the AG and DAG groups treated with macaíba almonds when compared to CONT and DG (Figure 3). Two-way ANOVA statistical analysis showed non-significant effects of dyslipidemia [F(1.44) = 0.8502, p = 0.3615], significant effects of macaíba consumption [F(1.44) = 21.26, p < 0.0001], and no interaction between dyslipidemia and macaíba consumption [F(1.44) = 0.8502 p = 0.8188].”

6. What is “Test-t” mentioned in the legends. Authors seem to mention Tukey’s test here. If it is so, eliminate “Test-t” from legends and write Tukey’s test completely.

Thank you, it was removed.

7. The article still need English language correction.
